# MARPE expander activation load with different configurations of extender arms heights: *in-vitro* evaluation

**DOI:** 10.1590/2177-6709.29.4.e242458.oar

**Published:** 2024-09-02

**Authors:** Staell Ribeiro de FARIA, Túlio Rodrigues de ANDRADE, Cristiane Barros ANDRÉ, Victor Angelo Martins MONTALLI, Jurandir Antonio BARBOSA, Roberta Tarkany BASTING

**Affiliations:** 1 Faculdade São Leopoldo Mandic, Dentistry Department (Campinas/SP, Brazil).; 2 Mogi das Cruzes University, Dentistry Department (Mogi das Cruzes/SP, Brazil).

**Keywords:** MARPE technique, Rapid maxillary expansion in adults, Stresses, Mini-implants, Técnica MARPE, Expansão rápida da maxila em adultos, Tensões, Mini-implantes

## Abstract

**Objective:**

Evaluate the load mini-implants exert on the artificial bone when expanding the MARPE EX in three different extension arm configurations.

**Methods:**

A device simulating the human palate was fabricated and attached to a universal testing machine, for conducting tests with different MARPE expanders (n=5): non-adjustable/control (MARPE SL, Peclab) or with low, intermediate, and high extender arms (MARPE EX, Peclab). The expanders were manually activated until failure of the device occurred, and maximum load values were recorded. Load averages were also calculated for every five activations until the twentieth activation.

**Results:**

The generalized linear mixed model for repeated measures over time showed that there was significant increase in load with activations for all expanders (*p*=0.0004). Up to the twentieth activation, the expander with low extender arms presented higher load than the others, while the expander with high extender arms showed lower load values (*p*<0.05). There was no significant difference among expanders regarding the number of activations (*p*=0.0586), although there was a trend towards fewer activations until fracture for the control expander. It was observed that the higher the configuration, the lower the force the mini-implants delivered to the bone. The control expander provided a force magnitude similar to that of the adjustable expander when positioned at the intermediate height.

**Conclusions:**

The activation load of MARPE expanders is influenced by the type of presentation of the extensor arms, with higher configurations resulting in lower force delivered by the mini-implants to the bone.

## RESUMO

**Objetivo:**

Avaliar a carga que os mini-implantes exercem em osso artificial, ao expandir o MARPE EX com três diferentes configurações de braços extensores.

**Métodos:**

Um dispositivo que simula o palato humano foi confeccionado e acoplado a uma máquina universal de ensaios, para a realização dos testes com os diferentes expansores MARPE (n=5): não ajustável/controle (expansor MARPE SL, Peclab) ou com braços extensores baixos, intermediários e altos (expansor MARPE EX, Peclab). Os expansores foram ativados manualmente até o momento que ocorreu a falha do dispositivo, registrando-se os valores máximos de carga. Também foram calculadas médias de carga para cada cinco ativações, até a vigésima ativação.

**Resultados:**

O modelo linear generalizado misto para medidas repetidas no tempo mostrou que houve aumento significativo da carga com ativações, para todos os expansores (*p*=0,0004). Até a vigésima ativação, o expansor com braços extensores baixos apresentou carga maior que os demais, enquanto o expansor com braços extensores altos apresentou carga menor que os demais (*p*<0,05). Não houve diferença significativa entre os expansores quanto ao número de ativações (*p*=0,0586), embora tenha havido uma tendência de menor número de ativações até a fratura para o expansor controle. Observou-se que, quanto mais alta a configuração, menor a força entregue pelos mini-implantes ao osso. O expansor controle forneceu magnitude de força semelhante ao expansor ajustável, quando posicionado na altura intermediária.

**Conclusões:**

A carga de ativação dos expansores MARPE é influenciada pelo tipo de *design* dos braços extensores, com configurações mais altas resultando em menor força aplicada pelos mini-implantes ao osso.

## INTRODUCTION

Maxillary transverse deficiency is a type of malocclusion that represents a skeletal problem characterized by the presence of transverse maxillary discrepancy, resulting in posterior crossbite, dental crowding, and a wider buccal corridor, which can impair smile esthetics.^1^ Moreover, maxillary transverse deficiency is recognized as a factor contributing to the development of Obstructive Sleep Apnea (OSA).^2^ It has a prevalence of 10% in the general population, with this type of issue occurring in 30% of patients seeking orthodontic treatment.^3^ ^,^ ^4^

Treatment of maxillary transverse deficiency involves opening the median palatal suture by separating the maxillary halves by means of rapid maxillary expansion (RME). This treatment is successfully applied in growing patients when the median palatal suture is not fully mature. However, in patients treated after the growth peak, the increased skeletal maturity of the median palatal suture causes resistance, thereby limiting the success of the technique.^5^ Therefore, many specialists consider that treatment of maxillary transverse deficiency involves surgical procedures such as surgically assisted rapid palatal expansion (SARPE), which are more invasive and costly.^6^

The introduction of mini-implants in Orthodontics is outstanding as one of the most significant changes in clinical practice.^7^ In 2010, the first case of RME using mini-implants was reported.^8^ This technique, called miniscrew-assisted rapid palatal expander (MARPE), was a treatment option for correcting maxillary atresia in adults.^9^ It involved an orthodontic expander anchored by mini-implants in the palatal and cortical bones of the nasal floor, so that its force was not directly delivered to the teeth but rather to the bone, avoiding the need for SARPE surgery.^10^ ^,^ ^11^

After the technique was launched in 2010, other different models with some variations were developed, and demonstrated effectiveness in the majority of cases.^12^ ^,^ ^13^ ^,^ ^14^ However, in patients with severe maxillary transverse discrepancy, limited results were observed because expansion can cause tissue damage to the lateral palatal mucosa.^15^ Therefore, a skeletal anchorage expander with individualized heights was developed.^16^ The aim of this new design, also known as suspended MARPE, was to expand maxillae with severe transverse discrepancies, deep and asymmetrical palates. This expander can be customized for each patient, with the help of four mini-implants and height adjustments.^16^ This new MARPE expander model has extender arms that allow the screw body to be positioned so that it does not collide with the lateral palatal mucosa.^17^ This expander exerts lower tension on the supporting teeth and enables stress to be distributed over the entire lateral lamina of the pterygoid process.^17^

Adult patients with severe transverse discrepancies have been observed to have greater limitations in executing the MARPE technique. Consequently, this may lead to a less favorable outcome due to the increased distance between the activation key and the palatal bone-supported mini-implants upon expander placement. Upon activation, this can cause inclination of the mini-implants, preventing the desired expansion. Therefore, during maxillary disjunction, the mechanical behavior of the mini-implants and the expander is of interest, especially due to the heavy forces applied to perform the above-mentioned procedure;^18^ and the clinician is responsible for selecting the ideal height of the extension arms, appropriate to the degree of transverse discrepancy of the maxilla of each patient.

Considering the different configurations and models of MARPE maxillary expanders demanded by the different anatomies of the patients, it is evident that the forces resulting from expansion activation are not always equal. Therefore, the aim of this study was to evaluate the load exerted by the mini-implants on artificial bone, when expanding the MARPE EX (which has individualized heights) in three different height configurations.

## MATERIAL AND METHODS

**Figure 1:**
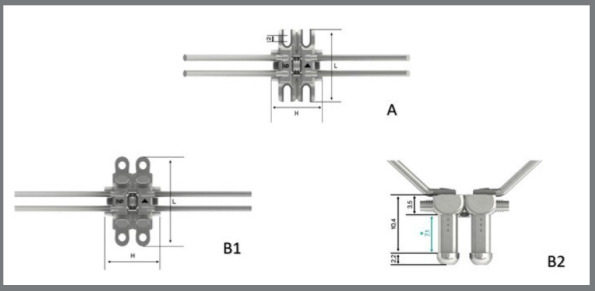
Expanders used in the study. A) Non-adjustable MARPE SL expander; B) Adjustable MARPE EX expander - (B1) top view, (B2) front view.

This was an experimental laboratory study. The experimental units consisted of expanders that were connected to artificial bone and fixed with self-drilling mini-implants (n=5). The factors under investigation were: types of expanders, including a non-adjustable control level (MARPE SL expander/Peclab, 11mm key) and three experimental height levels, namely low, intermediate, and high (MARPE EX expander/Peclab, 13mm key), as depicted in Figure 1; and the number of activations at four levels, grouped into every five activations, with tests continuing until maximum activation was reached and ending the test when there was fracture of the mini-implants and/or activation key or expander.

**Figure 2:**
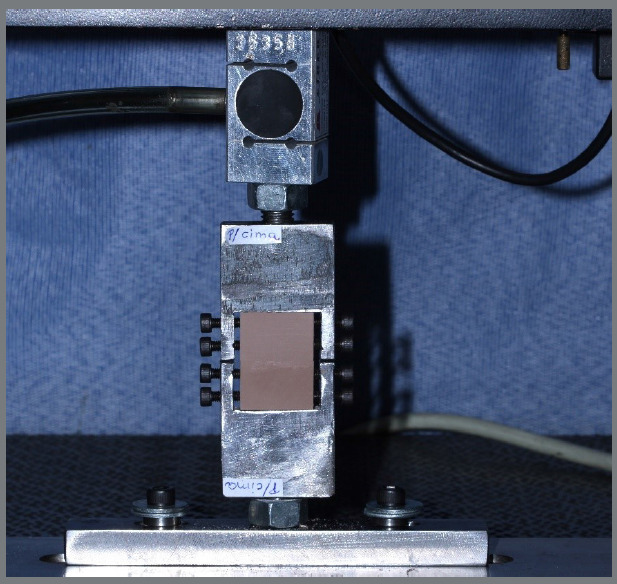
Bone blocks attached to the slots of the metal tips, by means of four screws each.

To simulate the human maxillary bone, 40 blocks of artificial polyurethane bone were used, with dimensions of 25.5 mm in length, 17 mm in width, and 6 mm in thickness, consisting of two rigid 1 mm thick surface layers representing the bone cortices. Their densities were type 4 (40 PCF) for cortical bone and type 3 (15 PCF) for medullary bone (Nacional Ossos, Jaú, SP). The artificial bone blocks were screw-retained to the channel of two metal tips, by means of four screws. The dimensions of these metal tips, made of steel were 39 mm in width, 47 mm in length, and 9.0 mm in thickness; and their channels dimensions were 16 mm in width, 30 mm in length, and 9.0 mm in thickness. For standardizing the fixation of the bone blocks in the channels, we started on one side of the tip with the screws positioned close to the inner edge of the channel. From this moment onwards, the screw was inserted with a magnitude of three turns. From then on, the bone blocks were positioned adjacent to these two screws and in the inner edge of the channel. The other two contralateral screws were inserted until they touched the bone block, at which point – turn was made on the screws, to stabilize the bone blocks (Fig 2).

**Figure 3:**
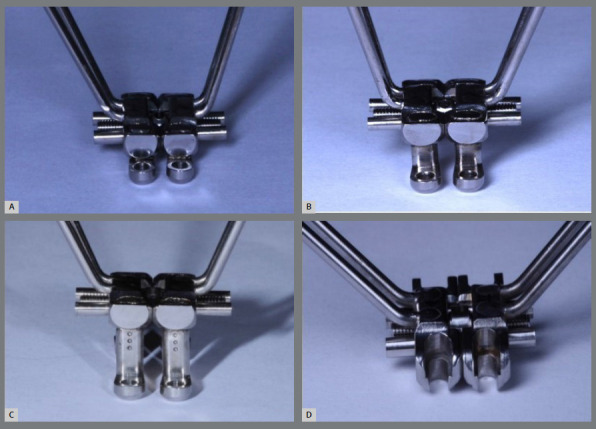
A) MARPE EX expander with low extender arms; B) MARPE EX expander with intermediate extender arms; C) MARPE EX expander with high extender arms; D) MARPE SL expander.

The EX expanders (experimental groups) had extender arms with a length of 12.60 mm. Considering this measurement, three different height configurations were determined: low position, at 2.20 mm (simulating a maxilla with mild transverse discrepancy, in which the expander key is closer to insertion of the mini-implants into the bone); intermediate position, at 6.70 mm (representing a maxilla with moderate transverse discrepancy, in which the expander key is in a median position relative to the insertion of the mini-implants into the bone); and high position, at 10 mm (simulating a maxilla with severe transverse discrepancy, in which the expander key is further from the insertion of the mini-implants into the bone). Only one configuration was performed for the SL expander (control group) because this expander is not adjustable (Fig 3). Laser welding was used to fix the EX extender arms in these three different positions. To ensure precise positioning of the arm heights, three guides (CAD/CAM) were made, one for each position.

A second guide was fabricated to standardize the placement of the MARPE expanders in the “metal tip/bone” assembly, with one guide for each configuration of the EX expander, and one guide for the SL expander. The welding of the EX expanders and the fabrication of the guides were carried out by Kika Digital Orthodontics (Sorocaba/SP, Brazil).

**Figure 4:**
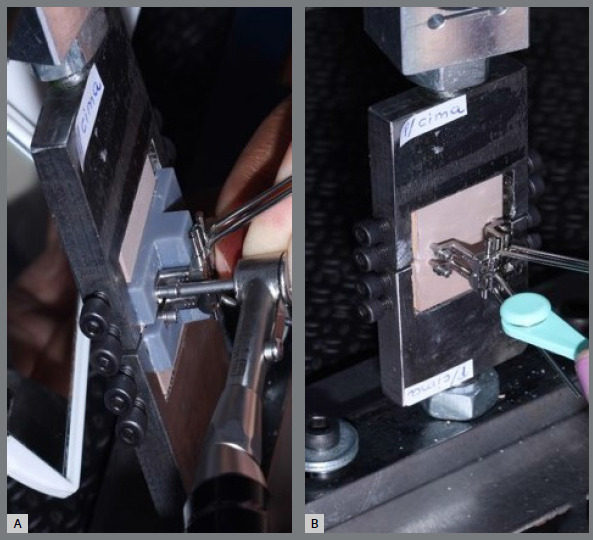
Installation sequence of the MARPE expander. A) Installation of mini-implants; B) Removal of the guides and start of activations.

Both expanders were positioned 2 mm away from the bone blocks, with the EX (expander arms) and SL (expander base) at this distance. The aim of using this positioning was to replicate intraoral conditions, which typically involve 1 to 2 mm of mucosa, and 1 to 2 mm is the ideal distance that the expander should be from this mucosa. After using the guide to position the expanders in the “metal tip/bone” assembly, fixed by four screws each, the cortical bone perforations were performed in the four holes. For this purpose, the cortical bone perforation drill from Peclab was used, driven by an Orthonia 010 electric motor (Jeil Medical Corporation, Guro-gu, Seoul, South Korea) with a maximum torque of 30 N when the electric motor stops operating. Subsequently, four 13-mm mini-implants, with a thread of 7mm and transmucosal portion of 6mm, were inserted, by using the medium MARPE insertion key from Peclab. The mini-implants were inserted perpendicularly and bicortically, also using the Orthonia 010 electric motor (Jeil Medical Corporation, Guro-gu, Seoul, South Korea) at 30 rpm. Finally, the guides were removed, and activations began (Fig 4). In the experimental group (MARPE EX), the anteroposterior distance between the mini-implants was 17.7 mm, and in the control group (MARPE SL), it was 13 mm. The mini-implants were inserted at a distance of 5.4 mm between the blocks, parallel in both groups. The two tips were positioned and connected to the universal testing machine.

For each type of expander and location, five repetitions were used (n=5). The mechanical tests were performed on a universal testing machine (EMIC DL2000, São José dos Pinhais, Paraná, Brazil), using a load cell of 200 kilogram-force (Kgf). In each test, the expander screw was manually activated by using the Peclab activation key, with intervals of 10 seconds, until the mini-implants, and/or activation key, or expander fractured.

After each mechanical test, the artificial bone blocks, MARPE, mini-implants, and activation key were replaced with new units. These devices were tested until maximum activation that caused fracture of the artificial bone or failure of the device, with the number of activations and the load value applied being recorded.

The mean loads were calculated for every five activations up to the twentieth activation. Initially, descriptive and exploratory data analyzes were carried out. In the exploratory analyzes, the normality of errors and homoscedasticity (homogeneity of variances) were assessed. As the data did not meet these necessary assumptions for applying a classic analysis of variance (ANOVA) with a general linear model, a mixed-effects generalized linear model for repeated measures over time was applied to analyze the load data over the course of the activations. In this model, the effects of “expander type”, “number of activations” and the interaction between them were considered. Generalized linear models were also estimated, considering the “expander effect” to analyze the total number of activations and the load in the last activation. All analyzes were performed using the R program, with a significance level of 5%.

## RESULTS

The results of load as a function of expander type and number of activations showed a significant increase in load with activations for all expanders (*p*=0.0004) (Table 1). There were differences between expander types (*p*=0.0135), but the interaction between the factors was not significant (*p*=0.0583). Up to the twentieth activation, the MARPE EX with low extender arms showed a higher load than the other expanders, and the MARPE EX with high extender arms showed a lower load than the others (*p*<0.05). Up to the fifteenth activation, the MARPE SL and MARPE EX with intermediate extender arms showed no significant difference in load between them (*p*>0.05).However, for activations 16 to 20, the MARPE SL showed a higher load than the MARPE EX with intermediate extender arms (*p*<0.05).

**Table 1:** Load (in Kgf) as a function of expander type and number of activations.

Expander	Number of activations			
	1-5	6-10	11-15	16-20
	Mean (SD)	Mean (SD)	Mean (SD)	Mean (SD)
MARPE SL	4.05 (0.77)^Db^	13.98 (1.76)^Cb^	19.29 (0.89)^Bb^	24.08 (1.10)^Ab^
MARPE EX with low extender arms	6.17 (1.25)^Da^	18.35 (1.19)^Ca^	26.69 (2.09)^Ba^	32.87 (2.94)^Aa^
MARPE EX with intermediate extender arms	3.51 (1.01)^Db^	12.16 (2.47)^Cb^	17.97 (2.32)^Bb^	21.49 (2.21)^Ac^
MARPE EX with high extender arms	1.78 (0.36)^Dc^	8.02 (1.25)^Cc^	13.54 (2.27)^Bc^	17.86 (2.09)^Ad^

Distinct superscript letters (capital letters horizontally and lowercase vertically) indicate statistically significant differences (p≤0.05). SD = standard deviation.

**Figure 5:**
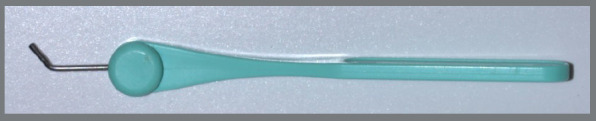
Activation key with a 45-degree bend of the active tip, demonstrating its deformation.

In all tests for all expanders, the tests were terminated because the activation key bent at a magnitude of 45 degrees (Fig 5).

There was no significant difference between the expanders relative to the number of activations (p=0.0586), despite a trend towards a lower number of activations until fracture for the MARPE SL expander (Table 2). However, at the last activation, the load was significantly higher in the MARPE EX with low extender arm, and lower in the MARPE EX with high extender arms (*p*<0.0001). There was no significant difference between the MARPE SL and MARPE EX with intermediate extender arms relative to load at the last activation (*p*>0.05).

**Table 2:** Number of activations until fracture and load (Kgf) in the last activation as a function of the expander.

Expander	Number of activations	Load at the last activation
	Mean (SD)	Mean (SD)
MARPE SL	28.40 (4.72)^a^	32.28 (4.12)^b^
MARPE EX with low extender arms	33.00 (6.40)^a^	41.28 (3.05)^a^
MARPE EX with intermediate extender arms	37.20 (7.36)^a^	29.36 (2.91)^b^
MARPE EX with high extender arms	39.00 (9.08)^a^	23.37 (2.93)^c^
p-value	0.0586	<0.0001

Different superscript letters vertically indicate statistically significant differences (p≤0.05). SD = standard deviation.

## DISCUSSION

The MARPE technique has been refined over time, with the use of new appliances. However, the mechanical behavior of mini-implants and the expander appliance during maxillary disjunction is of interest, especially due to the heavy forces applied during the procedure.^19^ Therefore, it has become increasingly important to understand this mechanical behavior of the expanders and their relationship with bone, in order to assist orthodontists in making clinical decisions.

The MARPE EX expander features a customizable configuration, thus expanding the indication of MARPE for patients with severe maxillary transverse discrepancies.^10^ It has been observed that this expander provides better conditions for distributing skeletal stress, with the distance between the palate and the appliance, and the distance between mini-implants appearing to be factors that allow for a wider distribution of forces along craniofacial structures.^17^ In the present study, it was observed that the forces resulting from expansion activation were not always equal, considering different expander configurations demanded by different maxillary anatomies. Thus, the results show that the MARPE EX with low extender arms presented the highest mean force load at the last activation, compared with the others; while the MARPE EX with high extender arms presented the lowest mean force load at the last activation, compared with the others. Despite the various variables and limitations present in the study - such as the absence of craniofacial structure resistance, the use of artificial bone, and being a laboratory study, without the factors related to the MARPE technique biomechanics -, it could be suggested that using the high extender arms position may lead to less expansion in maxillae with severe transverse discrepancy. Therefore, the MARPE EX with high extender arms may be a better recommendation for expanding maxillae with thinner bone thickness, in addition to using a more cautious activation protocol. In contrast, the MARPE EX with low extender arms - which delivered a higher force load at the last activation, compared with the other expanders - may be more recommended for expanding maxillae with greater bone thickness and a heavier activation protocol. If used for maxillae with thinner bone thickness, the recommendation is to alter the activation protocol by reducing the number of activations and, consequently, the load applied, and/or allowing a longer interval between activations, for load dissipation.

With each activation, it was found that there was a significant increase in load for all expanders. Moreover, up to the fifteenth activation, the MARPE SL and MARPE EX with intermediate extender arms did not show significant differences in load. This suggests that they could be used in maxillae with the same bone thickness. For better adaptation to the palate, the choice between them would depend on the amount of maxillary transverse discrepancy. In contrast, when comparing the MARPE EX with low extender arms and the MARPE SL expander, which are commonly chosen for expanding maxillae with mild transverse discrepancy, it was observed that the MARPE EX with low extender arms delivered a higher force (Table 1). A study carried out by Walter et al.^20^ with expanders without dental anchorage showed that they were capable of developing forces of up to 150 N. In the present study, the MARPE EX with low extender arms was able to produce the highest load in the last activation (41.28 kgf), which is equivalent to 404.5 N. This demonstrates that the force delivered to the bone with this MARPE EX design is much higher when compared to expanders without dental anchorage.

In all tests, failure of the expander occurred at the activation key, and no fracture/deformation of mini-implants or expansion screw was observed. According to Brunetto et al.,^9^ the deformation of MARPE anchorage mini-implants was associated with the distance of the force applied to the cortical/mini-implant interface, meaning that the further the expander screw was from the palate, the higher would be the probability of deformation.

It is important to note that, in the present study, no bone fracture or loosening of the mini-implants was observed. This may be related to the fact that the artificial bone had a medullary bone thickness of 4 mm and was bicortically enveloped in a 1-mm thick layer, totaling 6 mm. Redzepagic-Vrazalica et al.^21^ observed that the success of mini-implant insertion largely depended on cortical and bone thickness, with the highest average pull-out force occurring in a cortical bone ≥ 0.68 mm with an average force of 252.12 N (or 25.69 kgf), and the lowest average pull-out force occurring in a cortical bone with thickness < 0.62 mm with an average force of 113.50 N (or 11.56 kgf). It is noteworthy that the present study showed that the average force at the last activation was higher than the value of 25 kgf in the MARPE SL, EX low, and EX intermediate expanders. This means that the force delivery of these expanders is greater than the pull-out force of the mini-implants, suggesting that the force delivered by the MARPE system is robust and greater than the bone can withstand. Therefore, it would be prudent to establish a new activation protocol, with fewer activations or longer intervals between activations, especially in delicate cases in which the bone is thin.

The forces exerted by the expander appliance are directly related to the degree of resistance exhibited by the craniofacial structures, and thus, the results of the present study may diverge from clinical outcomes, due to various biomechanical factors related to the MARPE technique. Although there is a need for further research to be conducted to complement the results, this study highlights the importance of the clinician selecting the ideal height of the extension arms, which should be appropriate to the degree of transverse discrepancy of the maxilla of each patient.

## CONCLUSION

The results of the mechanical testing simulation indicate that the activation load of MARPE expanders is influenced by the type of presentation of the extensor arms, with higher configurations resulting in lower force delivered by the mini-implants to the bone.
